# TongueCaps: An Improved Capsule Network Model for Multi-Classification of Tongue Color

**DOI:** 10.3390/diagnostics12030653

**Published:** 2022-03-08

**Authors:** Jinghong Ni, Zhuangzhi Yan, Jiehui Jiang

**Affiliations:** 1 School of Communication and Information Engineering, Shanghai University, Shanghai 200444, China; njh123@shu.edu.cn; 2 School of Life Science, Shanghai University, Shanghai 200444, China; jiangjiehui@shu.edu.cn

**Keywords:** tongue color, capsule network, deep learning

## Abstract

Tongue color is an important part of tongue diagnosis. The change of tongue color is affected by pathological state of body, blood rheology, and other factors. Therefore, physicians can understand a patient’s condition by observing tongue color. Currently, most studies use machine learning, which is time consuming and labor intensive. Other studies use deep learning based on convolutional neural network (CNN), but the affine transformation of CNN is less robust and easily loses the spatial relationship between features. Recently, Capsule Networks (CapsNet) have been proposed to overcome these problems. In our work, CapsNet is used for tongue color research for the first time, and improved model TongueCaps is proposed, which combines the advantage of CapsNet and residual block structure to achieve end to end tongue color classification. We conduct experiments on 1371 tongue images; TongueCaps achieve accuracy is 0.8456, sensitivity is 0.8474, and specificity is 0.9586. In addition, the size of TongueCaps is 8.11 M, and FLOPs is 1,335,342, which are smaller than CNN in comparison models. Experiments have confirmed that the CapsNet can be used for tongue color research, and improved model TongueCaps, in this paper, is superior to other comparison models in terms of accuracy, specificity and sensitivity, computational complexity, and size of model.

## 1. Introduction

Tongue diagnosis has been recorded as early as in the classic Chinese medicine book ‘Huangdi Neijing’, which was to diagnose disease by observing the characteristics of tongue, and then, it made rapid progress in the diagnosis of exogenous fever. Nowadays, tongue diagnosis has become a unique diagnostic method under the guidance of Traditional Chinese Medicine (TCM) theory [1]. The content of tongue examination is divided into two parts: observation of tongue texture and tongue coating. Tongue color is an important content of tongue texture, and is generally divided into five categories: light red, red, deep red, light white, cyan [2]. Different colors can reflect different physiological and pathological states, blood rheology [2], and the attributes of pathogens [3], which is an important basis for effective clinical diagnosis, guidance of medication, efficacy judgment, and prognosis [2,4]. In addition, there are many studies on the analysis of the relationship between disease and tongue color [5,6,7,8], such as quantitatively analyzed tongue color in the case of blood stasis syndrome and non-blood stasis syndrome [5], which found the value of B chromaticity component of tongue color in breast cancer patients is greater than the R chromaticity component and the G chromaticity component [6] and quantitatively analyzed the tongue color of patients with rheumatoid arthritis [7,8]. Therefore, correct identification of tongue color is of great clinical significance.

With the development of computer-aided diagnosis technology, more and more researchers are committed to using computers to classify tongue color with image processing technology in order to improve objectification and classification accuracy. Most studies are based on machine learning [5,6,7,8,9,10,11,12,13,14,15,16,17]. In these studies, the researchers will first remove tongue coating as much as possible, leaving only tongue part as the research object, because tongue texture and tongue coating are staggered and distributed on the tongue [2], which makes the tongue coating have a certain influence on analysis of tongue color. There are two ways to separate tongue texture and tongue coating: one is to manually select pixels of tongue texture [5,6,9,10], and the other is to use clustering [11,12,13,14]. Then, they found that different methods of separation will obtain different results. In addition, the illumination of data acquisition environment used in these studies is not the same, which has a certain impact on results. Yang XY et al. reviewed and summarized 22 studies on tongue color classification, using CIE Lab and CIELCH color space to uniformly convert tongue color chromaticity values into L value, a value, b value, C value, and H value, and then found that there is an overlapping area in chromaticity value ranges between different tongue color, as well as no clear classification boundary, which makes it difficult to select one of them as the standard [15].

At present, deep neural network models are gradually being widely used in the field of image recognition because they can automatically extract complex features through the training of large amounts of data, without manual intervention, and have better prediction results for unknown data [18]. The most common framework in deep learning is convolutional neural network (CNN) [19]. Tang, Y.P. et al. used CNN, combined with multi-task learning, for tongue feature classification, and the final total accuracy rate reached 0.96 [20]. Another model framework, the CapsNet proposed by Professor Hinton [21], which not only greatly reduces the size of mode, but also makes more effective use of spatial location information, and it better encodes the relationship between local information and global goal. In some studies, it has been gradually confirmed to have better performance on classification tasks, which are of a limited number and low resolution datasets [22,23,24,25,26] compared to some CNN models, such as AlexNet [27], NDCNN [28], and NPMIL [29].

In conclusion, the above mentioned methods, based on machine learning, need to separate tongue texture and tongue coating first and then, manually select features, which is cumbersome and time consuming. We hope to reduce manual intervention via deep learning, letting the model automatically learn to separate tongue texture and tongue coating, as well as extract features related to tongue color to achieve end to end classification of tongue color. In addition, since CapsNet can make full use of spatial features, we choose CapsNet as our base model. In our work, we propose TongueCaps based on CapsNet. We will introduce the framework of our model and test performance of the model on our tongue color RGB images with different size and then, compare our model with CapsNet and some models based on CNN.

## 2. Materials and Methods

In this study, we proposed TongueCaps based on CapsNet and then, compared the performance of this model with other common CNN models on the tongue color classification task through experiments. In this section, we present the materials and methods used in the study, including data acquisition, data preprocessing, data expansion, framework of TongueCaps and comparison models, hyperparameter tuning methods in model training, evaluation matrices, and experimental platforms.

### 2.1. Data Acquisition

**Figure 1:**
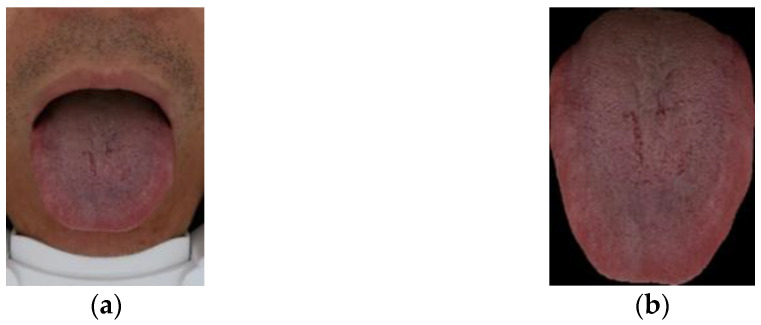
The acquired original image and the corresponding segmented image. (**a**) Raw image; (**b**) Image after segmentation.

The raw images used in this study were provided by Tianjin Huiyigu Technology Co., Ltd. Image data is acquired by tongue image acquisition equipment. When using the device to shoot, the face needs to be close to the device to reduce the impact of ambient light and then, stretch out tongue as much as possible to allow the device to capture the tongue. Figure 1a is an example of raw image, which is a 24-bit RGB image and includes two types of size: 1640 × 2460 and 1480 × 2220. Figure 1b is an image containing only tongue, which is obtained by using machine to automatically segment raw image, in order to remove the influence of lip color, face color, etc., on tongue color recognition.

**Figure 2:**
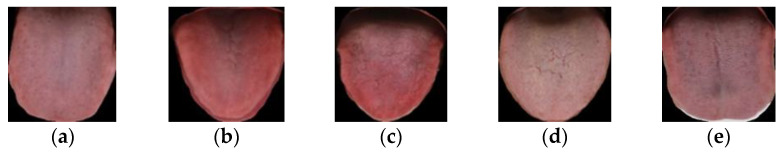
Samples of five types of tongue images. (**a**) Light red tongue; (**b**) Red tongue; (**c**) Deep red tongue; (**d**) Light white tongue; (**e**) Cyan tongue.

There are a total of 1371 cases in experiment data, including five categories: light red tongue, red tongue, deep red tongue, light white tongue, and cyan tongue. The image sample of each type of tongue color is shown in Figure 2.

The number of tongue color images in the five categories is shown in Table 1, 382 cases of light red tongue, 312 cases of red tongue, 104 cases of deep red tongue, 304 cases of light white tongue, and 269 cases of cyan tongue.

### 2.2. Data Reprocessing and Data Division

The first preprocessing is to uniform the size of the image to 128 × 128. The size of the tongue image data is different. For the convenience of experiment, the image size is fixed to uniform size by using equal scaling. First, reduce the long side of the image to the specified size, then reduce the short side with same proportion, and finally, fill the short side with black pixels to the same size as long side, the procedure as shown in Table 2.

The second preprocessing is color space conversion. The original image is in RGB color space because RGB color space generally does not reflect specific color information of the object very well, so we choose the HSV color space that is more commonly used in image processing. This operation can be achieved by using color space conversion function in the python package cv2.

Each type of tongue color in the data set is divided into training set and test set at 8:2 division situation, as shown in Table 3.

**Figure 3:**
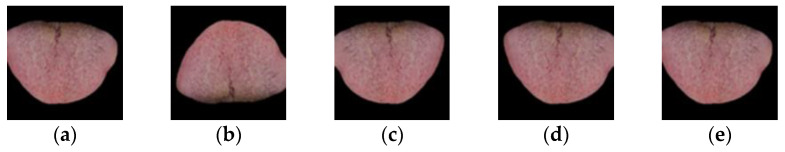
Data augmentation. (**a**) Original image; (**b**) Rotate 90 degrees; (**c**) Rotate 180 degrees; (**d**) Horizontal movement; (**e**) Vertical movement.

Before the experiment, the training data is expended by the methods of horizontal movement, rotation, vertical movement, and brightness adjustment. First three methods can be implemented by OpenCV package. Figure 3 is the sample of different ways of data expansion, Figure 3a is the original image, Figure 3b,c are obtained by rotating the original image by 90 degrees and 180 degrees, respectively, Figure 3d is obtained by moving the target horizontally in the original image, and Figure 3e is obtained by moving the target vertically in the original image.

Image brightness adjustment is implemented using Equation (1), *I*(*x*,*y*) and the pixel value at position (*x*,*y*) on the original image, *I′(x,y)* is the pixel value after adjusting the brightness, *k* is the adjustment factor, and the range of values is [0.25, 4]. When *k* < 1, the image brightness becomes brighter. When *k* > 1, the image brightness becomes darker.

(1) $$I^{\prime }\left(x,y\right)={\left(\frac{I\left(x,y\right)}{255}\right)}^{k}*255$$

**Figure 4:**
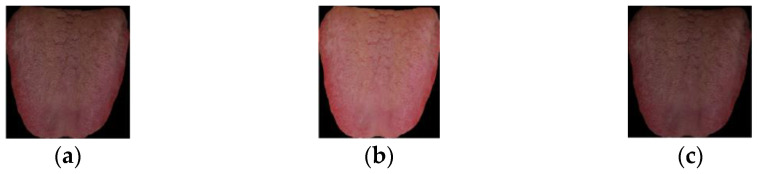
This is the tongue image in three different brightness levels. (**a**) Original image; (**b**) tongue when brightness adjustment factor *k* = 0.5; (**c**) tongue when brightness adjustment factor *k* = 1.5.

In our experiment, we choose k to be 0.5 and 1.5, and the processing result image is shown in Figure 4.

Finally, after data expansion, the data numbers of five class tongue color are basically balanced. The number of training sets is as follows: light red 976 cases, red 1000 cases, deep red 924 cases, light white 980 cases, cyan 1032 cases, and 4912 cases in total.

### 2.3. Model: TongueCaps

**Figure 5:**
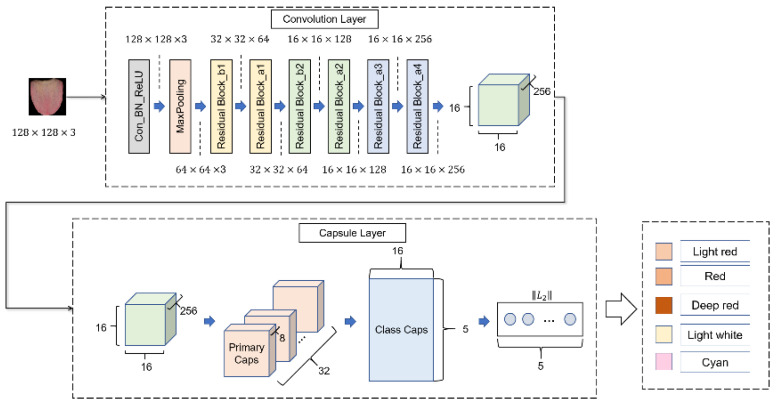
This is the framework of TongueCaps. It is composed of two parts, the convolution layer and the capsule layer, which input an image through these two parts and finally, output the probability that the input image belongs to five kinds of tongue colors.

The framework of TongueCaps is shown in Figure 5, including two main layers: convolution layer and capsule layer. The convolution layer is used to extract features, and we consider the problem of gradient disappearance that easily occurs in deep neural networks, so we adopt a shortcut connection structure in residual block [28]. The capsule layer includes Primary Caps and Class Caps. The function of capsule layer is to effectively combine the extracted features for final classification. In the following, we will introduce, in detail, the structure of convolution layer, the structure of capsule layer, and the parameter update method of capsule layer: dynamic routing algorithm.

When the size of the input image is 128 × 128, the parameters in each layer of TongueCaps are shown in Table 4, and the following describes the numbers in the parameters column. In the 1–8 lines, the first number represents the number of convolution kernels, the second parameter represents the size of the convolution kernel, and the third parameter represents the stride size. The parameters in the 9th line represent 8 groups of convolution operations. The number of convolution kernels in each group of convolution operations is 32, the size of the convolution kernel is 3 × 3, and the stride size is 1.

In the following content, we will introduce the model framework in detail, including the structure of each layer of the model, the calculation principle of forward propagation of the model, the update method of weight coefficient, and the loss function of the model.

#### 2.3.1. Convolution Layer

**Figure 6:**
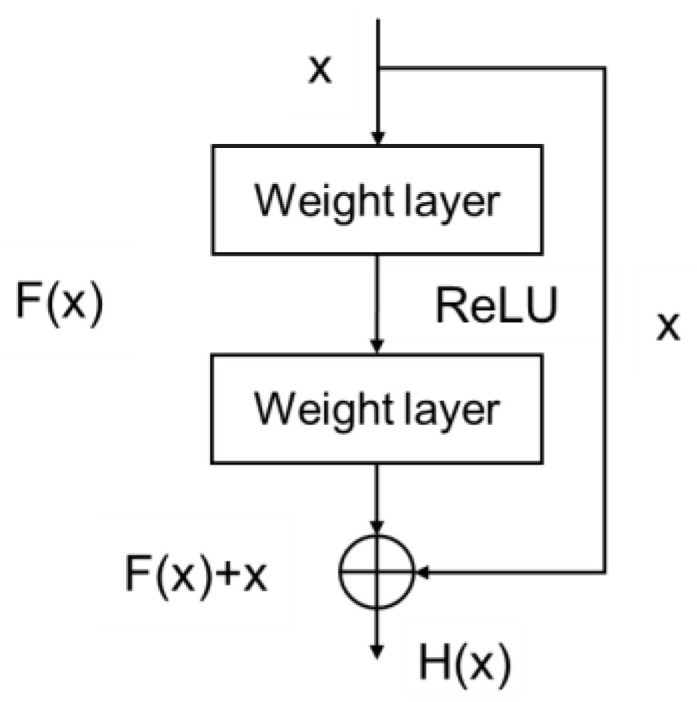
The structure of residual block.

The convolution layer of the initial capsule network has only one layer. For complex images, the feature extraction ability of one convolution layer is weak, and the extraction of low level features such as edges cannot fully learn the semantic information of the image. With an increasing number of convolution layers, the level of extracted features can be enriched [30], so we decided to increase the convolutional layer to enhance the feature extraction ability of the model. However, as the number of convolutional layers increases, the deeper the model becomes, and the more likely it is that the gradient disappears during the model training process, which hinders training of models. Therefore, we decided to use shortcut connection structure of residual block [31] in our convolution layer. The structure is shown in Figure 6.

**Figure 7:**
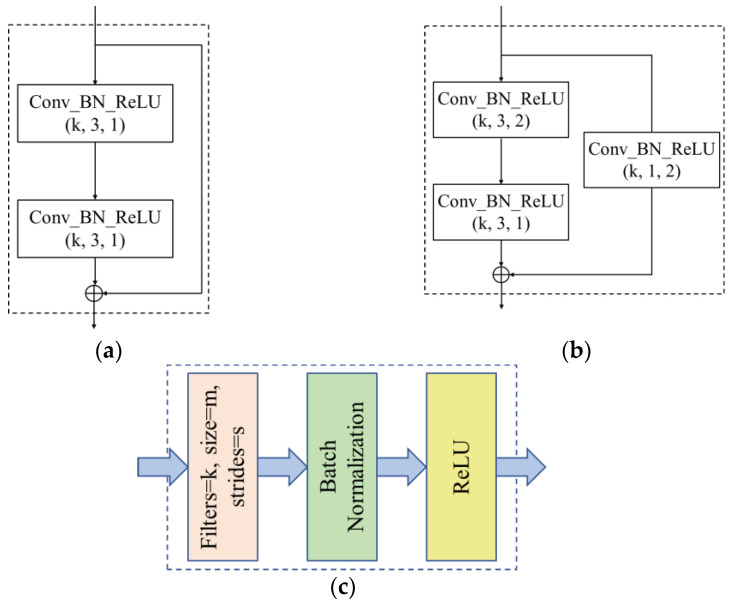
Two types of residual block used in TongueCaps. (**a**) Residual Block_a; (**b**) Residual Block_b; (**c**) Conv_BN_ReLU (k, m, s). The difference between residual blocks_a and b is whether one of the two branches needs to convolve the input. Conv_BN_ReLU is composed of a convolutional layer, a batchnormalization layer, and a ReLU activation layer.

When multiple layers are stacked, the output is not only a simple direct mapping F(x) that satisfies the input x, but it also satisfies another mapping: H(x) − x. Simply put, the input of a latter layer is the addition of the output of a previous layer and the input x of a previous layer: H(x) = F(x) + x. The residual block structure we used has two specific forms, residual block a and residual block b, as shown in Figure 7a,b, in addition, Conv_BN_ReLU (k, m, s) is shown in Figure 7c, and the bracket indicates the parameters of convolution layers, meaning that the number of convolution kernels is k, kernel size is m × m, strides is s, and convolution form takes type ‘same’. BN is Batch Normalization layer, and ReLU is nonlinear activation layer.

#### 2.3.2. Primary Caps

**Figure 8:**
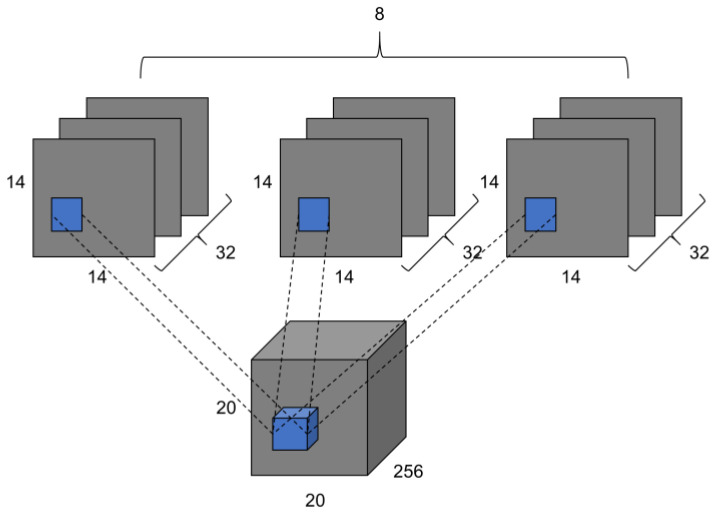
Convolution of Primary Caps.

The function of the Primary capsule layer is to map the output obtained by the convolutional layer into a vector as the input vector of Class Caps. Primary Caps consists of two parts of operation: convolution and capsule. For example, the size of the input feature maps is 256 × 16 × 16 (channels, width, height), the Primary Caps has 8 units, every unit has 32 channels convolutional layer, and every convolutional layer with 3 × 3 kernel size has strides 1. Then, the output of all channels feature maps with a size of 14 × 14 × 32, and output is 8 × 14 × 14 × 32, as shown in Figure 8. Every channel converts feature maps to a vector, so the output of Primary Caps are 8 vectors, with dimensions of each vector as 14 × 14 × 32 = 6272.

#### 2.3.3. Class Caps

**Figure 9:**
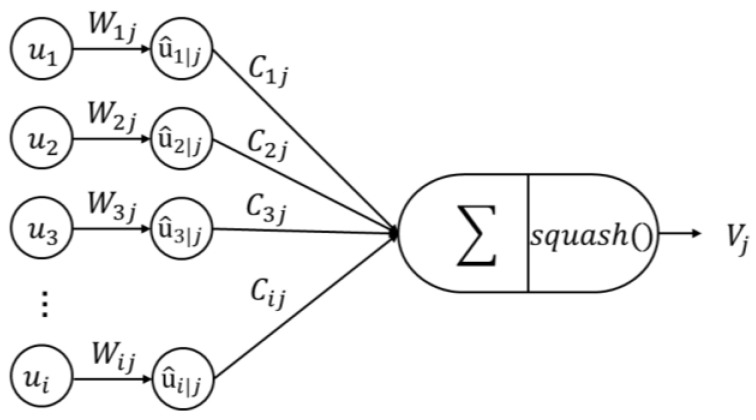
The figure of Capsule.

Class Caps receives the output vector from the Primary Caps layer. Output of Class Caps are k vectors, and k is the number of tongue the color category. In the above example, Primary Caps finally outputs 8 vectors. Then, the number of input vectors of Class Caps is 8, and each vector is the input vector of a capsule unit. Each capsule unit corresponds to an ‘entity’ of the image, the direction of output vector represents attribute of the ‘entity’, and the length of output vector represents probability of the ‘entity’ existing on the image. The capsule unit calculates the output through forward propagation. Moreover, the input of the capsule unit is a vector, and the output of capsule unit is also a vector. The following describes how forward propagation process of the capsule is realized. Figure 9 shows the structure of a capsule, $u_{i}$ is the output of capsule i in layer l, and $u_{j}$ is the output of capsule j in layer (l+1). First, transform the input, as shown in Equation (2), multiply the $u_{i}$ by the weight $w_{ij}$ to get the prediction vector $\;{\hat{u}}_{j|i}$.

(2) $$\;{\hat{u}}_{j|i}=W_{ij}u_{i}$$

Then, perform a weighted sum on the prediction vector, according to Equation (3), to get $s_{j}$. Among them, $c_{ij}$ is the weight coefficient, which is updated by the dynamic routing algorithm.

(3) $$s_{j}=\underset{i}{\sum }c_{ij}\;{\hat{u}}_{j|i}$$

Finally, use Equation (4) to operate nonlinear activation and ensure the length of output $V_{j}$ in the interval [0, 1].

(4) $$V_{j}=\frac{\|s_{j}\|{}^{2}}{1+\left\|s_{j}\right\|{}^{2}}\frac{s_{j}}{\left\|s_{j}\right\|}$$

#### 2.3.4. ‖*L*_2_‖ Layer

This is the last layer of TongueCaps, receiving k vectors from Class Caps, where k is the number of tongue color category. This layer uses Equation (5) to calculate the length of each vector. Among it, n means dimension of vector, and $x_{1}$ represents the value of each dimension. Length of each vector represents probability that image belongs to each tongue color category, and finally, it determines tongue color category of input image as the tongue color category corresponding to vector that length is maximum.

(5) $$Length=\sqrt{x_{1}{}^{2}+x_{2}{}^{2}+\ldots +x_{n}{}^{2}}\;$$

#### 2.3.5. Dynamic Routing Algorithm

**Figure 10:**
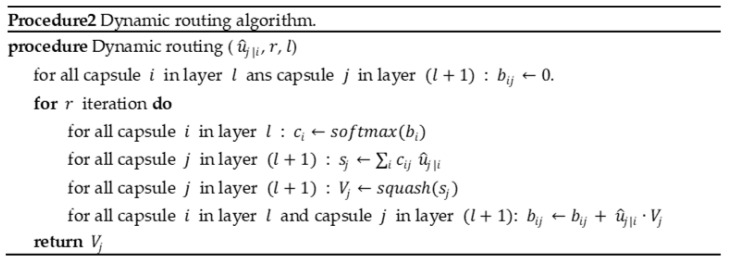
Procedure of Dynamic routing algorithm.

When the capsule forward propagation was introduced in the previous section, it was mentioned that $c_{ij}$ is updated through dynamic routing algorithm. This algorithm is introduced in Figure 10, and among them, $w_{ij}$ is affine transform matrix, which is updated by back propagation, $b_{ij}$ in the table is a parameter that needs to be initialized, and then, use Equation (6) to calculate $c_{ij}$,

(6) $$softamax\left(b_{i}\right):\;c_{ij}=\frac{\exp\left(b_{ij}\right)}{\sum_{j}\exp\left(b_{ij}\right)}$$

In fact, the direction of the update of the weight $c_{ij}$ is to give a large weight to the output vector of the capsule neuron in the layer l that has a large contribution to the final recognition.

#### 2.3.6. Loss Function

The capsule network uses the vector length to represent the probability of the existence of each entity. When the entity appears in the image, it is hoped that the loss will be small, and when the entity does not exist, it is hoped that the loss will be large, so the marginal loss is used in Equation (7),

(7) $$L_{k}=T_{k}\max{\left(0,m^{+}-\|V_{k}\|\right)}^{2}+\lambda \left(1-T_{k}\right)\max{\left(0,\|V_{k}\|-m^{-}\right)}^{2}$$

Among them, $T_{k}$ is the classification indicator function (class *k* exists, the value is 1, otherwise it is 0); $V_{k}$ is the output vector of the net; $m^{+}$ is used to punish false positives, the value is 0.9; $m^{-}$ is used for false negatives, and the value is 0.1; λ is the proportional coefficient, adjust the proportion of the two punitive, the value is 0.5.

### 2.4. Experimental Comparison Model

**Figure 11:**
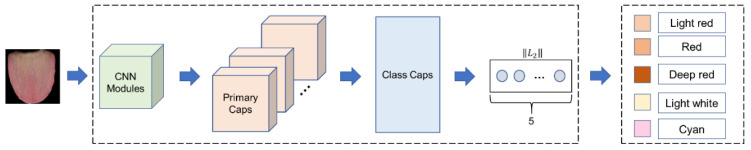
The framework of CNN + Caps.

The base model is CapsNet [21]. Other comparison models include VGG16, ResNet18, ResNet50, ResNet101, Inception V3, and CNN + Caps. Where CNN + Caps is as shown in Figure 11, the CNN module selects two forms: the convolution module in literature [22] and the convolution module in the TongueCaps model, which removes the shortcut connection structure for the convenience of the following description. The former is recorded as CNN1 + Caps, and the latter is recorded as CNN2 + Caps.

### 2.5. Model Training

First is to determine hyperparameters of model training. The hyperparameters tuned in this experiment include learning rate, optimizer, and batchsize. The learning rate value is (0.1, 0.001, 0.0001), the optimizers are SGD and Adam, and the batchsize value is (8, 16, 32, 64, 128). Then, use the method of grid search to choose the value of hyperparameters. When each setting determines the learning rate, optimizer, and batchsize, use five-fold cross validation to calculate the average accuracy of the 5 models on the validation set. Then, use the hyperparameter corresponding to the highest average accuracy as the final hyperparameter value to train the entire training set.

### 2.6. Metrices

Our work uses the accuracy, sensitivity, and specificity to evaluate the performance of the model. In the calculation of three assessment criteria, as shown in Equations (8)–(10),

(8) $$Accuracy=\frac{TP+TN}{TP+TN+FP+FN}$$

(9) $$Sensitivity=\frac{TP}{TP+FN}$$

(10) $$Specificity=\frac{TN}{TN+FP}$$

where *TP* is the number of positive samples correctly predicted by the model, *TN* is the number of negative samples correctly predicted by the model, *FP* is the number of positive samples incorrectly predicted by the model, *FN* is the number of negative samples incorrectly predicted by the model. For multi-classification, take one of the classes as positive samples and the rest as negative samples. From this, the accuracy, specificity, and sensitivity of each class can be calculated, and the average can be calculated to evaluate the overall performance of the model. In addition, we also use training time, size, and FLOPs to evaluate the model. Model size represents the size of parameters, and FLOP represent the computational complexity of the model.

### 2.7. Experimental Platform

The operating system used in this experiment is the Ubuntu 16.04 system, and the integrated development environment is PyCharm 2020.2. The deep learning framework is tensorflow1.13 and keras2.2.4. The version of other packages are as follow: python3.6, h5py2.10.0, numpy1.19.5, scipy1.5.4, opencv-python4.5.4, and matplotlib3.3.4. The hardware central processing unit used in this experiment is Intel (R) Core (TM) i9-9900K CPU at 3.6 GHz, the running memory is 16.00 GB, and the image processor is GeForce RTX 2080.

## 3. Results

**Figure 12:**
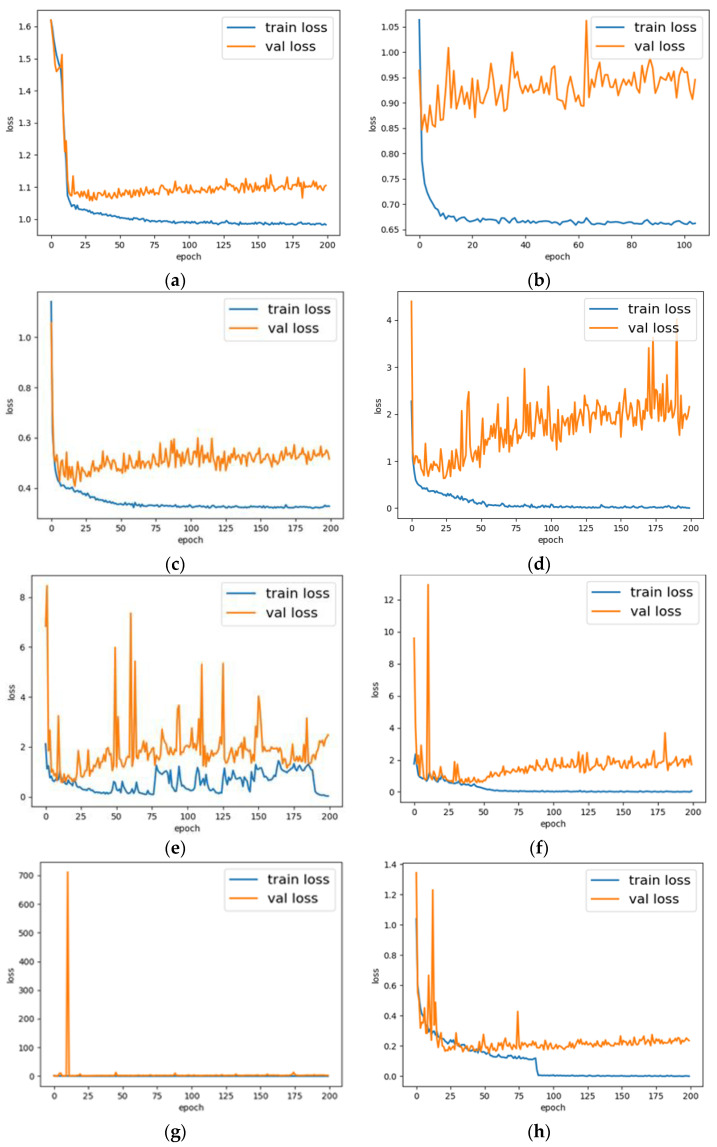
The loss curve of each model. (**a**) CapsNet; (**b**) CNN1 + Caps; (**c**) CNN2 + Caps; (**d**) VGG16; (**e**) ResNet18; (**f**) ResNet50; (**g**) ResNet101; (**h**) TongueCaps.

The loss curve of each model are shown in Figure 12. The figure includes the loss curve of the training set and the loss curve of the validation set, which are represented by the blue curve and the orange curve, respectively. The horizontal axis represents the numbers of training epoch, and the vertical axis means the loss of the train and validation set. In Figure 12, Figure 12a is the loss curve of CapsNet, Figure 12b is the loss curve of CNN1 + Caps, Figure 12c is the loss curve of CNN2 + Caps, Figure 12d is the loss curve of VGG16, Figure 12e–g are the loss curve of ResNet18, ResNet50, and ResNet101 respectively, and Figure 12h is the loss curve of TongueCaps.

In addition, after model testing is performed using a data set containing different brightness, while the accuracy, sensitivity, and specificity are obtained by calculating the confusion matrix of each model. The results are shown in Table 5. In addition, the training time, size, and FLOPs of each model are also given in Table 5.

Judging from the loss curve, the model proposed in this paper can achieve better convergence during training, and the final loss is close to the limit. From the results, it can be seen, compared to the other models, TongueCaps shows the highest accuracy, sensitivity, and specificity, while having a small size and FLOPs.

## 4. Discussion

From the loss curve and test results in Figure 11, the original CapsNet exhibits an underfitting situation. The possible reason is that the CapsNet has only one convolutional layer, thus for tongue images that are more complex than digital images, the ability to extract features is weak. In addition, the two models of CNN + Caps both increase the number of convolutional layers on the basis of the CapsNet. From the perspective of the loss curve, the problem of underfitting is alleviated to a certain extent, but there is still a problem of overfitting. The possible reason is that the complexity of the model cannot match the complexity of the image and the limitation of the amount of data. Therefore, the residual block structure is added on the basis of CNN2 + Caps to increase the complexity of the model and relieve the problem of underfitting and overfitting. From the comparison of Figure 12c,h, it is shown that, after residual block structure is added to the CNN2 + Caps, the TongueCaps can effectively alleviate the phenomenon of overfitting. In addition, TongueCaps performed better than other models based on CNN, the possible reason is that CNN will lose the characteristics of spatial location information [32] due to the pooling layer, while CapsNet can learn spatial features between local and global through a dynamic routing algorithm. Moreover, from the Table 5 it can be seen, compared to the other CNN models, TongueCaps has the smallest model parameters. The main reason is that, compared with convolution layer, Primary Caps and Class Caps have reduced a lot of parameters. Primary Caps compresses input features into vector, and the Class Caps uses forward propagation of the capsule to obtain output from the input vector, which has reduced many parameters.

## 5. Conclusions and Future Work

In this paper, we used the Capsule Network for tongue color classification, for the first time, to realize the end to end classification of light red tongue, red tongue, deep red tongue, light white tongue, and cyan tongue. Furthermore, we improved the Capsule Network and proposed TongueCaps, which combine the advantages of both Residual block and Capsule network. The model has the advantages of both the capsule network and the residual block structure. Our model inherits the original characteristics of the capsule network, is more robust to image affine transformation, is suitable for small sample data sets, and retains the spatial relationship between features. In addition, our model has been improved in the feature extraction stage, increasing the number of convolutional layers and introducing a residual block structure to alleviate the over fitting phenomenon and enhance the model’s ability to extract features. The proposed model is verified through experiments, and the classification performance of the capsule network model in tongue color classification is improved. In addition, compared with the more commonly used CNN models, such as VGG16, ResNet18, etc., it can obtain higher accuracy, specificity, and sensitivity, and at the same time, it can also maintain a small model size and a low model complexity, which provides the possibility for the subsequent realization of the high performance and light weight of the tongue color classification model. Due to the small size of the model, the follow-up work can be to deploy the model to the mobile terminal or the web terminal, and then, input the image to be tested and output the tongue color result, to provide a basis for clinical tongue diagnosis and facilitate the realization of portable tongue diagnosis. However, the features related to tongue color that are extracted by this model are poor in interpretability, and further research can be done in this area.

## Figures and Tables

**Table 1:** Sample image data statistics.

Tongue Color Type	Light Red	Red	Deep Red	Light White	Cyan
Image number	382	312	104	304	269

**Table 2:** Procedure of image size unification.

**Procedure 1** Image Size Unification.
**procedure** Unification ($\;Image_{h\times w}$, L) Step 1: import cv2 Step 2: calculate the scaling factor $k=\frac{L}{h}$ Step 3: cv2.resize ($\;Image_{h\times w}$, L, w×k) Step 4: use black pixels on short side to fill length w×k to length L **return** $\;Image_{L\times L}$

^1^ Image is the image to be processed, *h* and *w* are the length of the long side and the length of the short side of the original image respectively, *L* is the size of the processed image.

**Table 3:** Division situation.

	Light Red	Red	Deep Red	Light White	Cyan
Train set	244	200	66	196	172
Val set	62	50	17	49	43
Test set	76	62	21	59	54
Total	382	312	104	304	269

**Table 4:** Parameters of TongueCaps.

Layer	Parameters	Output Shape
Con_BN_ReLU	3, 7 × 7, 1	128 × 128 × 3
MaxPooling	3, 3 × 3, 2	64 × 64 × 3
Residual Block_b1	64, 3 × 3, 2	32 × 32 × 64
Residual Block_a1	128, 3 × 3, 1	32 × 32 × 64
Residual Block_b2	128, 3 × 3, 2	16 × 16 × 128
Residual Block_a2	128, 3 × 3, 1	16 × 16 × 128
Residual Block_a3	256, 3 × 3, 1	16 × 16 × 256
Residual Block_a4	256, 3 × 3, 1	16 × 16 × 256
Primary Caps	32, 3 × 3, 1, units = 8	8 × 6272

**Table 5:** Results of each model.

Model	Accuracy	Sensitivity	Specificity	Time	Size	FLOPs
CapsNet	0.2108	0.2181	0.8086	20 h 13 min	322.21 KB	51,340
CNN1 + Caps	0.2569	0.2580	0.8184	3 h 55 min	2.09 MB	1,249,882
CNN2 + Caps ResNet18	0.4130 0.7537	0.3227 0.7597	0.8390 0.9365	28 h 22 min 10 h 8 min	12.84 MB 134.29 MB	1,307,322 23,439,481
ResNet50	0.7316	0.7438	0.9309	5 h 52 min	306.58 MB	53,317,922
ResNet101	0.7573	0.7761	0.9387	8 h 15 min	525.34 MB	91,302,331
VGG16	0.7255	0.7622	0.9306	29 h 14 min	385.05 MB	67,231,786
TongueCaps	0.8456	0.8474	0.9586	5 h 45 min	8.11 MB	1,335,342

## Data Availability

The data that support the findings of this study are available from Tianjin Huiyigu Technology Co., Ltd (Tianjin, China). Restrictions apply to the availability of these data, which were used under license for this study. Data are available from Tianjin Huiyigu Technology Co., Ltd. with the permission of Tianjin Huiyigu Technology Co., Ltd. The data cannot be shared at this time as the data form part of an ongoing study.
